# Stable Self‐Floating Reduced Graphene Oxide Hydrogel Membrane for High Rate of Solar Vapor Evaporation under 1 sun

**DOI:** 10.1002/gch2.202000053

**Published:** 2020-09-28

**Authors:** Pengyu Zhuang, Duo Li, Ning Xu, Xiaoqiang Yu, Lin Zhou

**Affiliations:** ^1^ National Laboratory of Solid State Microstructures College of Engineering and Applied Sciences School of Physics Key Laboratory of Intelligent Optical Sensing and Integration Ministry of Education Nanjing University Nanjing 210093 P. R. China; ^2^ School of Physics Southeast University Nanjing 211189 P. R. China; ^3^ Nanjing Xiaozhuang University Nanjing 211171 P. R. China

**Keywords:** interfacial hydrothermal assembly, rGO hydrogel membranes, solar desalination, solar vapor generation, solar water purification

## Abstract

Highly efficient vapor generation with considerable stability under natural solar irradiance is a promising technology for seawater desalination and wastewater purification. Here a broadband solar absorber of reduced graphene oxide hydrogel membrane (rGOHM), synthesized via an environmentally friendly one‐step hydrothermal reduction process, is demonstrated, which shows a high rate of solar vapor production and superior stability. The porous rGOHM containing more than 99.5% water within its small volume floats on the surface of water, exhibiting efficient solar absorption of **≈**98% across 300–2500 nm, as well as sufficient water‐pumping pathways. The evaporation rate can be tuned by changing the water volume. By controlling the water volume, the self‐floating rGOHM can enable efficient interfacial solar vapor generation at a high rate of **≈**2.33 kg m^−2^ h^−1^ under 1 sun, which is comparable to the rate generated by the evaporator with an extra insulator. In addition, the evaporation rate of rGOHM is only slightly affected at a high saltwater concentration (at least 15 wt%), and the rGOHM shows mechanical and physical stability. The superior evaporation performance combined with efficient eradication of wastewater contaminants, cost‐effectiveness, and straightforward fabrication process, makes this rGOHMs ideal for advanced high‐concentration seawater desalination and wastewater treatment technologies.

## Graphical abstract

A self‐floating reduced graphene oxide hydrogel membrane (rGOHM) is demonstrated for stable, high‐performing solar vapor evaporation under 1 sun. The rGOHM shows an interfacial solar vapor generation rate of 2.33 kg m^−2^ h^−1^, which is comparable to the rate generated by an evaporator with an extra insulator and is only slightly degraded in highly concentrated brine up to 15 wt%.

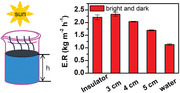

## 1 Introduction

Water and energy are inextricably linked in economic activities of humanity. The energy–water nexus is exacerbated by the shortage of both fresh water resources and energy generation infrastructure.^[^ ^1^, ^2^ ^]^ Advanced solar vapor generation enabled by rational designed nanomaterials is becoming one of the most promising technologies due to the abundance of free and clean solar energy as well as the emergent advances of interfacial heat localization,^[^ ^3^, ^4^, ^5^, ^6^ ^]^ inspiring the revival of solar‐thermal‐based applications in desalination,^[^ ^7^ ^]^ water purification,^[^ ^8^ ^]^ chemical separation,^[^ ^9^ ^]^ sterilization,^[^ ^10^, ^11^ ^]^ and electricity power generation,^[^ ^12^ ^]^ etc. Up to now, tremendous efforts have been made by designing and fabricating a variety of photothermal materials such as metallic nanoparticles,^[^ ^7^, ^13^, ^14^, ^15^, ^16^, ^17^ ^]^ nanowires,^[^ ^18^ ^]^ and carbon‐based materials including graphene oxide (GO),^[^ ^3^ ^]^ graphene,^[^ ^19^, ^20^, ^21^ ^]^ reduced graphene oxide (rGO),^[^ ^5^, ^22^ ^]^ graphite,^[^ ^6^, ^23^ ^]^ carbon nanotubes,^[^ ^24^, ^25^ ^]^ and carbonized wood.^[^ ^26^, ^27^, ^28^ ^]^ However, among various photothermal materials, the high‐efficiency solar vapor generations were mostly performed by using costly concentrators or extra insulators, which increases the cost and/or complexity of the entire devices^[^ ^19^, ^21^, ^23^ ^]^ and the stabilities of solar absorbers have rarely been studied. Therefore, the systems of highly stable and cost‐effective solar absorbers combined with high solar vapor generation efficiency need to be developed for practical application.

In order to enable a simultaneous energy and mass transfer process, an ideal solar evaporator for 1 sun vapor generation is required at least four crucial components, which includes efficient and broadband solar absorption, low thermal energy loss, proper solar system structure, and sufficient water supply as well as timely vapor escape.^[^ ^23^, ^29^ ^]^ So far, some efforts have been made on improving the evaporation rates under 1 sun, with relatively high evaporation rates (>1.6 kg m^−2^ h^−1^) and high solar thermal conversion efficiencies (>80%), especially, by using carbon‐based solar absorbers.^[^ ^20^, ^21^, ^30^, ^31^, ^32^, ^33^, ^34^, ^35^ ^]^ For instance, Zhu and co‐workers prepared an rGO‐based aerogel modified with sodium alginate and carbon nanotubes by directly freeze‐drying the GO mixture, and then by thermal reduction, which showed a rate of 1.62 kg m^−2^ h^−1^ with an efficiency of 83% under 1 sun.^[^ ^36^ ^]^ Hu et al. fabricated a jellyfish‐like solar absorber consisting of carbon black/GO composite layer with aligned GO pillars as water‐pumping ways by 3D printing technique, and obtained a rate of 1.27 kg m^−2^ h^−1^ with an efficiency of 87.5% under 1 sun.^[^ ^37^ ^]^ Wang and co‐workers fabricated a bilayer rGO film on a polystyrene foam by combining bar casting, thermal and chemical reduction, which generated an evaporation rate of 1.31 kg m^−2^ h^−1^ with an efficiency of 83% under 1 sun.^[^ ^5^ ^]^ Qu and co‐workers achieved an evaporation rate of 1.62 kg m^−2^ h^−1^ with a solar thermal conversion efficiency of 86.5% by using long‐range vertically aligned graphene sheets membrane as an absorber.^[^ ^20^ ^]^ Liu and co‐workers obtained an evaporation rate of ≈1.7 kg m^−2^ h^−1^ with an efficiency of > 90% by using hierarchical graphene foam deposited on the substrate of porous Ni foam.^[^ ^21^ ^]^ Although the efficiencies of the above solar absorbers have been improved to a high value, the water supply may not be provided enough during evaporations, causing the low evaporation rates.

Recently, the evaporation rates were improved further by utilizing hydrogels, which were made from polymers^[^ ^30^ ^]^ or polymer‐based mixtures with a few quantity of reduced graphene oxide or carbon as additives^[^ ^38^, ^39^ ^]^ and then freeze‐dried. However, the hydrogel with only absorbers of graphene‐based materials has not been realized, and it still lacks a facile but effective method to prepare hydrogel membranes with no need of complicated treatment, and the stabilities of hydrogels have also rarely been studied. So, there is still room to develop a facile but versatile method to prepare graphene‐based absorbers with high solar vapor generation rate and stabilities, more cost effectiveness.

In this report, we first fabricated reduced graphene oxide hydrogel membranes (rGOHMs) directly by a one‐step and environment‐friendly hydrothermal reduction method, which can be a facile method to synthesize graphene‐based hydrogel membranes. Compared to the reported graphene‐based materials,^[^ ^20^, ^21^ ^]^ which need energy‐intensive thermal treatment at 1000 °C to increase their absorbance and then etched by plasma to increase their hydrophilicity,^[^ ^20^, ^21^ ^]^ our rGOHM shows a highly efficient and broadband solar absorption (≈98%) without further treatment. The rGOHM contains more than 99.5% water within its small volume and can self‐float on water surface. The continuous water supply and vapor escape are ensured by the micrometric, nanometric, and sub‐nanometric pores, and nanocapillaries of self‐assembled rGOHMs.^[^ ^40^, ^41^, ^42^ ^]^ In addition, the superior stability of rGOHM makes sure the stable evaporation performance.

## 2 Results and Discussion

### 2.1 Synthesis of rGOHM

**Figure 1:**
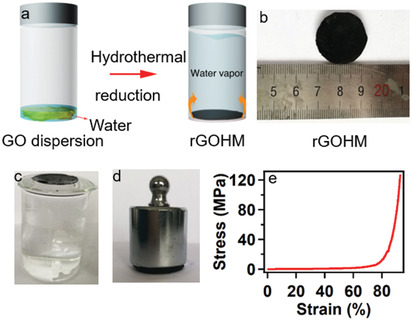
The fabrication and properties of rGOHM. a) The schematic of the fabrication process of rGOHM by hydrothermal reduction method. b) The synthesized rGOHM. c) Digital image of a prepared rGOHM supporting weight without deformation. d) Compressive stress–strain curve of one rGO hydrogel. e) The rGOHM floating on water surface.

The procedure of fabricating rGOHMs via the one‐step hydrothermal reaction is shown in **Figure** **1**a. The entire thermal reduction was processed in a Teflon‐lined autoclave at 120 °C for 12 h without adding any extra reduction agents (see more details in the “Experimental Section”). In this method, the volume of GO dispersion was controlled. Thus, the rGO sheets can assemble into thin films at the water/vapor interface (Figure 1a) instead of water bulk to prepare graphene monoliths.^[^ ^43^ ^]^ The rGOHM prepared with this method is totally black in appearance (Figure 1b), which is beneficial for efficient light absorption. During the hydrothermal process, the overheated supercritical water played the role of a reducing agent, which partially removed the functional groups of GO sheets and recovered their aromatic structures, significantly enhancing the attraction force between adjacent rGO sheets via strong π–π conjugating interactions.^[^ ^43^, ^44^ ^]^ Since the rGO sheets are flexible, many π‐stacking sites were generated between them, forming cross‐linkers in rGOHM that behave as bridges between thin‐stacked rGO sheet layers by partially overlapped or coalesced rGO sheets.^[^ ^43^ ^]^ The cross‐linking of rGO sheets results in the excellent mechanical strength and physical stability of the rGOHM (Figure 1c,d; Movie S1, Supporting Information). Detailed experiment revealed that the rGO hydrogel possesses a compression strength of 1.4 MPa and a strain of 91% at the break (Figure 1d), which are even comparable to the compression strength and strain of the dried rGO aerogel with high elasticity.^[^ ^45^ ^]^ The water adsorption of rGOHM (ratio of adsorbed water mass to total mass of rGO aerogel and adsorbed water) was analyzed by floating the rGO aerogel on water surface, which was prepared by freeze‐drying the rGOHM. The water adsorption of rGO aerogel reached a balanced state within 5 s, with a value of ≈95.5 wt% (Figure S1, Supporting Information). The extremely high water content and fast water adsorption of rGOHM make sure that there is enough water supplied instantaneously during solar evaporation. Although the rGOHM has a high water content within bulk, when put on water, it still can self‐float on the water surface (Figure 1e), enabling the interfacial localization of heat within rGOHM.^[^ ^6^ ^]^

### 2.2 Structure Characterization of rGOHM

**Figure 2:**
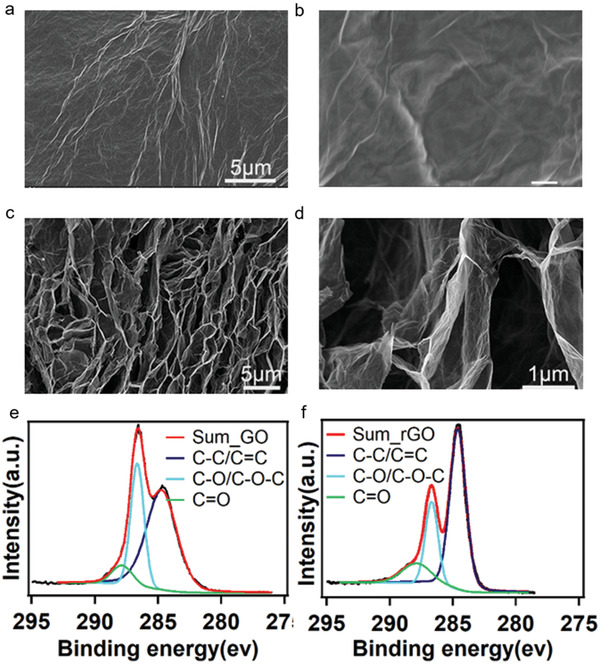
The morphologies, structures, and chemical compositions of the rGO aerogel membrane. a,b) SEM images of surface morphologies of freeze‐dried rGO aerogel membrane at low and high resolutions. c,d) SEM images of cross‐sectional morphologies and structures of freeze‐dried rGO aerogel membrane at low and high resolutions. e) C1s spectrum of GO aerogel membrane. f) C1s spectrum of rGO aerogel membrane.

The morphologies and structures of the rGO aerogel obtained by freeze‐drying rGOHM are characterized by scanning electron microscopy (SEM, FEI Helios Nanolab 600i) (**Figure** **2**a–d). As illustrated in the top view of SEM image (Figure 2a,b), there are many distinct wrinkles on the surface but no observable pores at the detected resolution of hundreds of nanometers (Figure 2b). However, intrinsic defects of nano‐ and sub‐nanopores exist on the surface of rGOHM.^[^ ^46^ ^]^ In the cross‐sectional SEM image (Figure 2c,d), a 3D framework was generated with interconnected pores in the range of sub‐micrometer and several micrometers, which are isolated by stacked thin layers of rGO sheets cross‐linked with each other.

The chemical compositions and functional groups of GO and rGO sheets before and after hydrothermal reaction are studied by X‐ray photoelectron spectroscopy (XPS). There are three fitting peaks occurred at the positions of 284.7, 286.6, and 287.9 eV for GO aerogel, which correspond to the chemical shifts of aromatic C—C/C=C, C—O/C—O—C, and C=O groups^[^ ^35^, ^47^ ^]^ (Figure 2e), accounting 62.5%, 30.5%, and 7%, respectively. After hydrothermal reduction, the above three peaks of rGO aerogel appeared at 284.6, 286.7, and 287.8 eV, respectively, almost the same positions as GO aerogel, showing 62.9% C—C/C=C, 23.9% C—O/C—O—C, and 13.2% C=O (Figure 2f). The ratio of C/O increased from 1.81 of GO to 2.53 of rGO (Figure S2, Supporting Information). The total content of C increased from 64.4% to 71.7%, indicating that the GO sheets were partially reduced. The residual functional groups are able to provide surface and bulk hydrophilicity to rGOHMs.

**Figure 3:**
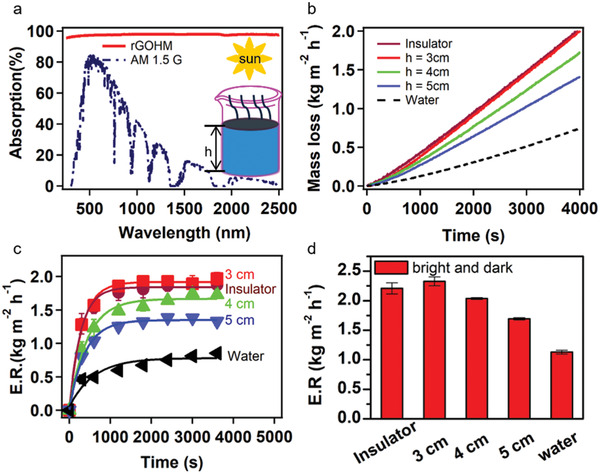
Solar steam generation performance of ≈2 mm thick rGOHMs. a) Solar absorption of rGOHM and the normalized spectral solar irradiance density of air mass 1.5 global (AM 1.5 G) tilt solar spectrum in blue dashed line. b) Mass losses of solar steam devices as a function of time under 1 sun (1 kW m^−2^ of solar flux) with rGOHM absorbers by using a polystyrene foam as an insulator, floating them on water with different volume heights of 3, 4, and 5 cm, and with pure water. c) The absolute evaporation rates of solar steam devices as a function of time. d) The total averaged evaporation rates of solar steam devices under 1 sun by considering the environmental contribution. The room temperature and humidity were kept at about 30 °C and 40%, respectively, during experiments.

The optical absorption performance of the solar absorber plays a crucial role in solar vapor generation. The solar absorption spectrum of rGOHM was measured with a ultraviolet–visible–near‐infrared spectrophotometer (UV 3600, Shimazu) combined with an integrating sphere accessory (ISR 3100), as shown in **Figure** **3**a. One can observe that, without modification with extra absorbing materials (such as carbon nanotubes and carbon black particles), the solar absorption of the rGOHM reached ≈98% in the entire solar spectrum (300–2500 nm), which is higher than the solar absorption of rGO‐based aerogel modified by adding carbon nanotubes,^[^ ^35^ ^]^ and has a similar absorption compared to the graphene‐based aerogels.^[^ ^19^, ^21^ ^]^

In order to reduce the heat conduction loss to bulk water, researchers have tried to use absorbers of low thermal conductivity,^[^ ^19^, ^36^ ^]^ steam generation devices consisting of bilayer structures using the bottom layer as a thermal insulator,^[^ ^5^, ^11^, ^22^, ^23^, ^26^, ^27^, ^48^ ^]^ or with extra insulators.^[^ ^3^, ^4^, ^20^, ^49^ ^]^ Herein, we tried to control the water volumes used to reduce conduction heat loss and also used an extra insulator as a comparison. Actually, the rGO aerogel has an extremely low thermal conductivity of 0.034 W m^−1^ K^−1^. It means that the framework materials contribute little to the overall thermal conductivity of the rGOHM, which may be on the order of that of pure water (0.58 W m^−1^ K).^[^ ^50^ ^]^

### 2.3 Solar Evaporation Performance

The evaporation rates of rGOHMs were systematically studied by floating the rGOHMs on water surface with different volumes of water and recording the mass losses of water under 1 sun irradiation (Figure 3b). To compare the evaporation rates with and without an extra insulator, the mass loss was also recorded by introducing a polystyrene foam as an insulator at the bottom of hydrogel and water pumps to supply water, similar to the method reported by the literature.^[^ ^3^ ^]^ The absolute evaporation rates versus time are calculated from the plots of the curves of absolute mass losses (with dark evaporation production subtracted from the total mass loss under 1 sun illumination), and their averages were also determined when the evaporation reached a quasi steady state by measuring three times (Figure 3c; Figure S3, Supporting Information). As can be seen from Figure 3c, the evaporation rate of the rGOHM with an insulator reached a high value (1.54 kg m^−2^ h^−1^, the thermal limit of photothermal vapor generation^[^ ^51^ ^]^) within 10 min, and become balanced within 20 min. The average absolute evaporation rate of rGOHM with an insulator reached 1.90 ± 0.09 kg m^−2^ h^−1^. Due to relatively small water volume of 3 cm height, the absolute evaporation rate of the self‐floating rGOHM shows a similar trend of evaporation and reached a quasi‐balanced state in 20 min (Figure 3b,c), showing an average absolute evaporation rate of 2.00 ± 0.07 kg m^−2^ h^−1^ (Figure S3, Supporting Information), completely comparable to the rate of rGOHM with an insulator. The evaporation rate was decreased with the increase in water volume. The absolute averaged evaporate rates of rGOHM self‐floating on water surface of 4 and 5 cm heights were decreased to 1.72 and 1.38 kg m^−2^ h^−1^, respectively. Even so, the absolute average evaporation rate of rGOHM floating on water with a height of 5 cm is 0.68 times higher than the evaporation rate of pure water with the same volume height. By considering the extra evaporation rates under dark environment, the total evaporation rates of rGOHMs with an extra insulator, self‐floating on water of 3, 4, and 5 cm heights, reached 2.21 ± 0.09, 2.33 ± 0.07, 2.04 ± 0.02, and 1.69 ± 0.02 kg m^−2^ h^−1^, respectively (Figure 3d). The evaporation rate of our rGOHM floating on water surface with a height of 3 cm was compared to the rates of a few representative graphene‐based aerogels and polymer‐based hydrogels with rGO, as summarized in Table S1 (Supporting Information). The evaporation rate of our rGOHM was much higher than the graphene‐based aerogels, and is among the few best values of hydrogels with graphene‐based materials as additives.

**Figure 4:**
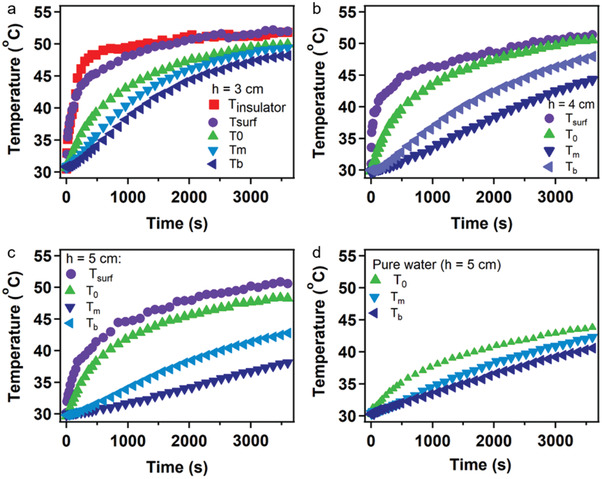
Temperature variations of solar steam generators at different positions as a function of time. a–d) Surface temperatures of rGOHM with/without insulator, and temperatures at surface, middle, and bottom positions of water of different heights. *T*_0_ refers to the surface temperature of water, *T*_m_ refers to the temperature at middle position of the water, and *T*_b_ refers to the bottom temperature of water.

To further clarify the evaporation rate change of the rGOHM as a function of time, the temperatures on top surface of rGOHM and at different positions of the water were monitored, which were recorded immediately under 1 sun irradiation during experiments. As can be seen from **Figure** **4**, the surface temperature of rGOHM with an insulator was fast increased to a high value of 49 °C in 10 min, then changed slightly with a balanced temperature of ≈52 °C. When the water volume of 3 cm height was used, the surface temperature of rGOHM without any insulator showed a similar trend with time, which was improved to ≈49 °C in 20 min, then increased to a balanced temperature of 52 °C. At the same time, the temperatures at different height positions of the bulk water increased fast within 30 min. After which, the temperatures reached a close value, only a few degrees lower than the surface temperature of the rGOHM. With water volume height being increased, although the surface temperatures of rGOHMs were only a few degrees lower than the surface temperature of rGOHM on water surface with a water volume of 3 cm, it took longer for the temperature in bulk water to reach a balanced state. The difference between the temperatures at the middle or bottom positions within bulk water and surface temperature of rGOHM increased with the increase in water volume, which means more conducted heat to water was lost. In addition to the lowered evaporation enthalpy of water in hydrogels,^[^ ^30^ ^]^ the higher temperature of bulk water also lowered the evaporation enthalpy. It should be the reason that the rate of rGOHM decreased with the increase in water volume.

It is worth noting that, although the evaporation rate is pronounced, we will not discuss the solar vapor conversion efficiency here mainly due to two primary concerns. On the one hand, the extremely high evaporation rate of rGOHM is partially attributed to the low evaporation enthalpy of water in hydrogels.^[^ ^30^, ^38^ ^]^ However, this value is not constant and cannot be calculated with a proper technology since it changes with temperature and the water content in the hydrogel. As a result, one can hardly calculate the solar vapor conversion efficiency precisely under such a complicated condition. As can be suggested from the thermograms of thermogravimetry (TG)–differential scanning calorimetry (DSC) in Figure S4 (Supporting Information), the water evaporates relatively slow before 80 °C, and its evaporation enthalpy correspondingly changed slowly. With temperature being increased further, water evaporation increased significantly, and its evaporation enthalpy changed, accordingly, largely. On the other hand, for most application circumstances such as solar desalination or water purification, one would like to employ the evaporation rate instead of the solar‐to‐vapor efficiency as the essential figure of merit for the overall evaluation.

**Figure 5:**
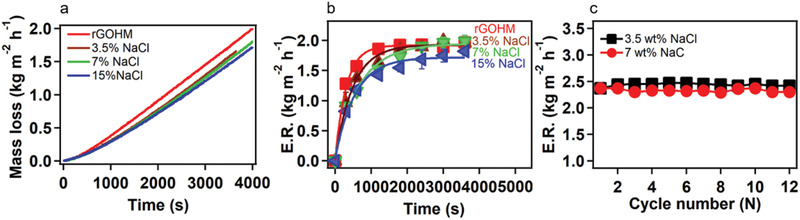
Solar steam generation performances of rGOHMs. a) Mass losses of solar steam devices as a function of time under 1 sun (1 kW m^−2^ of solar flux) with water and NaCl solutions of different concentrations. b) The absolute average evaporation rates of solar steam devices as a function of time. c) The evaporation rates of solar steam devices at balanced state under 1 sun with 5% and 7% NaCl solutions as a function of time.

The solar evaporation experiments of rGOHM were also conducted at high NaCl concentrations to study their solar performances. As shown in **Figure** **5**a,b, it takes a bit longer to reach balanced states with the increase in salt concentration. However, the steady‐state evaporation rates changed a little. At the steady state, the absolute evaporation rates of rGOHMs with 3.5% and 7% of NaCl solutions both reached 2.00 kg m^−2^ h^−1^, the same as the value of floating rGOHM on water surface. However, the rate with 15% NaCl solution reached 1.8 kg m^−2^ h^−1^, only a bit lower than the rate with water. By considering the dark evaporation, their corresponding total evaporation rates reached 2.33 and 2.17 kg m^−2^ h^−1^, respectively (Figure S5, Supporting Information). Besides, the balanced evaporation rates of rGOHM changed a little with time at the measured salt concentrations (Figure 5c), showing a durability for salt–water evaporation, which is superior to practical applications such as high‐concentration brine desalination and wastewater purification.

### 2.4 Stability of rGOHM

**Figure 6:**
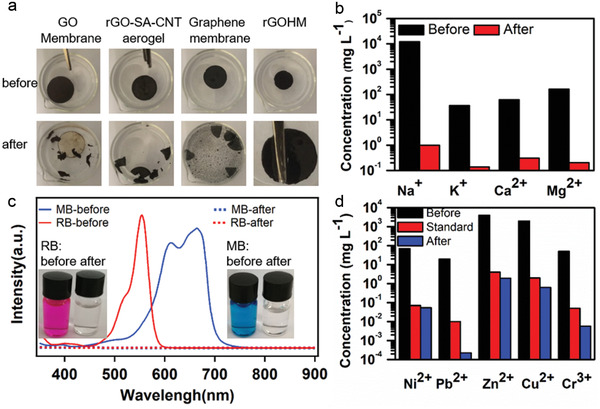
The stability of several carbon‐based absorbers and the performances of rGOHM in desalination and wastewater purification. a) The physical states of GO membrane, rGO‐SA‐CNT aerogel, graphene membrane, and rGOHM before and after shaking with a tweezer prepared with similar methods as in refs. ^[^ ^3^, ^20^, ^36^ ^]^. b) Concentrations of four primary ions of seawater before and after solar desalination. c) The UV absorption of wastewater containing simulated dyes of methylene blue (MB) and rhodamine‐B (RB) (20 mg L^−1^) before and after solar thermal purification. d) The ion concentrations of heavy metals in wastewater before and after solar thermal purification, and the standard concentration of ions defined by WHO.

The physical stability of the solar absorbers is also critically important for practical operation in harsh circumstances. In **Figure** **6**a, we demonstrated the stability test of our rGOHM as well as several representative carbon‐based absorbers of GO membrane, rGO‐SA‐CNT aerogel and graphene membrane, prepared with similar methods as described in refs. ^[^ ^3^, ^20^, ^36^ ^]^. After shaking the absorbers with a tweezer for about 2 min in water (Movies S2–S4, Supporting Information), the GO, rGO‐SA‐CNT aerogel, and graphene membrane were fractured into small pieces (Figure 6a), while our rGOHM remains intact even after strongly stirring in water for several minutes with a small stirring bar (Movies S5 and S6, Supporting Information).

### 2.5 Desalination and Wastewater Purification

The effects of the rGOHMs for desalination and wastewater purifications were evaluated by using simulated seawater with 3.5 wt% seasalt, as well as wastewaters containing organic dyes and heavy metals. As shown in Figure 6b, after solar desalination with 3.5 wt% saltwater, the concentrations of four primary ions of Na^+^, Mg^2+^, Ca^2+^, and K^+^ were reduced distinctly, showing a Na‐ion rejection of 99.99%. The concentration of Na^+^ ions is below the salinity levels defined by the World Health Organization (WHO) and the standard of the US Environmental Protection Agency (EPA).^[^ ^52^ ^]^ After purifying the wastewater containing methylene blue (MB) and rhodamine‐B (RB) of 20 mg L^−1^, the collected water from steam is clear and showing no absorption peaks of UV light (Figure 6c), indicating that all the dyes are rejected. While for wastewater polluted by heavy metals, the concentrations of the heavy metal ions of Ni^2+^, Pb^2+^, Zn^2+^, Cu^2+^, and Cr^3+^ are decreased by at least three orders of magnitude compared to its original concentration after purification, which is lower than the standard concentration defined by WHO (Figure 6d). The results suggest that the solar vapor generator of our rGOHM can effectively desalinate the seawater and purify the wastewater for practical applications.

## 3 Conclusion

In conclusion, rGOHMs as the most cost‐effective carbon‐based solar absorbers were first synthesized via a facile and environment‐friendly hydrothermal method without adding any chemical agents. The extremely high evaporation rates were realized due to the properties of high water content (>99.5 wt%), continuous water supply (>95.5 wt%), broadband solar absorption (≈98%) of hydrogels, and the reduced evaporation enthalpy of water in rGOHM. The balanced evaporation rate of self‐floating rGOHM on water can be tuned by changing the water volumes, which can be increased by decreasing water volume. The rate reached ≈2.33 kg m^−2^ h^−1^ under 1 sun, and changed a little with salt concentration, which could completely compete the rate with an extra insulator. In addition, our rGOHM shows a durability of solar vapor evaporation and superior mechanical and physical stabilities. The pronounced solar vapor production rate combined with superior physical and chemical stabilities, cost‐effectiveness, as well as straightforward material fabrication process enables our rGOHM as an ideal candidate for advanced solar water purification, especially for target water sources with a high concentration of salts or contaminants, serving as a powerful supporting strategy apart from filtration‐based technologies claiming on low‐concentration cases.

## 4 Experimental Section

##### Materials

Graphite powder (≈40 µm) was obtained from Qingdao Henglide Graphite Co., Ltd. H_2_SO_4_ (98%), P_2_O_5_, KMnO_4_, HCl (36%), H_2_O_2_, NiCl_2_·6H_2_O, PbCl_2_, ZnCl_2_, Cu(NO_3_)_2_·3H_2_O, and CrCl_3_·3H_2_O were purchased from Sinopharm Chemical Reagent Co., Ltd., and used without further treatment. K_2_S_2_O_8_ was obtained from Shanghai Aladdin Reagent Co., Ltd. Methylene blue and rhodamine‐B were purchased from Shanghai Macklin Biochemical Co., Ltd.

##### Synthesis of GO

GO was synthesized from natural graphite powders by a modified Hummers’ method.^[^ ^53^, ^54^, ^55^, ^56^ ^]^ In detail, 10 g of graphite powder (40 µm) was added into a 250 mL bake containing a mixture of H_2_SO_4_ (40 mL), K_2_S_2_O_8_ (8.4 g), and P_2_O_5_ (8.4 g) under stirring at 80 °C. The mixture was kept for 4.5 h at 80 °C under vigorously stirring. After cooling to room temperature, deionized water was added. When temperature was cooled down, the diluted mixture was vacuum‐filtered with a sand core funnel having pores in the range of 16–40 µm and washed with deionized water. After drying at room temperature, preoxidized graphite was obtained. Then the obtained graphite was added to a 1000 mL bake containing chilled 98% H_2_SO_4_ (230 mL) in an ice‐water mixture bath. After stirring for 0.5 h, KMnO_4_ (60 g) was added slowly under continuous stirring at a temperature lower than 10 °C. After stirring for another 0.5 h, the temperature was increased to 35 °C, and the mixture was kept for 2 h under stirring. Then, the brown paste generated was slowly added to 2 L cooled water under stirring. Following that, 30% H_2_O_2_ was added dropwise until no bubbles were appeared. Then, 2 L deionized water was further added, and the mixture showed a color of brilliant yellow. The mixture was left undisturbed for 2 days, and then the nearly clear supernatant was poured out. The precipitated mixture was repeatedly washed with water and centrifuged successively with 1 m HCl solution for three centrifugation cycles to remove residual metal oxides and then washed with deionized water until the decantate became neutral. The brown solids were obtained by centrifugation and then freeze‐dried to get GO sponge.

##### Preparation of rGOHMs

A GO suspension of 5 mg mL^−1^ was prepared by dispersing the synthesized GO in water and stirring it for 10 h. After that ≈3.5 mL of GO suspension was poured into a 50 mL Teflon line, sealed in an autoclave. The rGOHM of ≈2 mm thickness was prepared by putting the autoclave in a vacuum oven at 120 °C for 12 h. Several samples of rGO aerogels used for measurements by SEM and XPS were fabricated by freezing the hydrogels in liquid nitrogen and freeze‐drying them for 40 h. The GO membrane for Raman and XPS measurements was prepared by spray coating GO suspension onto a paper substrate and freeze‐drying it.

##### Preparation of Simulated Seawater and Polluted Water

The salt solutions of 3.5, 7, and 15 wt% concentrations for saltwater evaporations were prepared by dissolving NaCl in water, and the simulated 3.5 wt% seawater for desalination was prepared by dissolving seasalt in water. The wastewater containing MB and RB was prepared by dissolving them in water with a concentration of 20 mg L^−1^. The simulated wastewater containing heavy metal ions of Ni^2+^, Pb^2+^, Zn^2+^, Cu^2+^, and Cr^3+^ was prepared by dissolving NiCl_2_·6H_2_O, PbCl_2_, ZnCl_2_, Cu(NO_3_)_2_·3H_2_O, and CrCl_3_·3H_2_O in water with concentrations of 70, 20, 4000, 2000, and 50 mg L^−1^, respectively, which were 1000 times of standard concentration of the ions except for Pb^2+^and 2000 times of standard concentration of Pb^2+^defined by WHO.

##### Characterizations

The morphology and structure of rGO aerogel were characterized by SEM (Dural‐beam FIB 235, FEI Helios Nanolab 600i). The surface chemical compositions of GO and rGO membranes were analyzed by XPS (Thermo Fisher Scientific Al Kα source). The optical transmittance and reflectance spectra of the rGOHM in the range of 200–2500 nm were measured by a ultraviolet–visible–near‐infrared spectrophotometer (UV–vis) equipped with an integrating sphere (weighted by air mass 1.5 global (AM 1.5 G) solar spectrum). Its absorption efficiency was calculated by *A* = 1 − *R* − *T*, where *R* and *T* represent the reflection and transmission efficiency, respectively. The thermal diffusivity of rGO aerogel was measured using Netzsch LFA 467 Nanoflash. The absorbance spectra of wastewater containing dyes and collected water were measured by a UV–vis spectrophotometer (Cary 500). The mass loss of rGOHM and its heat flow were studied by heating rGOHM in an uncovered Al_2_O_3_ pan from 30 to 200 °C at a rate of 5 K min^−1^ with TG and DSC (SAT 449F3, Netzsch). Concentrations of ions in brine and collected clean water were tracked by inductively coupled plasma spectroscopy (inductively coupled plasma optical emission spectrometer, Optima 5300 DV, PerkinElmer Instrument). Tensile measurement was performed by an electronic universal testing machine (RGWT‐400‐20) equipped with a 20 N load cell. The gauge length was ≈8 mm, and the loading rate was set as 0.5 mm min^−1^. The water adsorption was defined as the mass ratio of adsorbed water in rGO aerogel membrane to the total mass of wet rGO aerogel membrane, measured by putting the rGO aerogel membrane directly on water surface and weighting the mass of wet rGO aerogel membrane as a function of time.

##### Experimental Setup for Solar Vapor Generation

The experiments of steam generation were conducted under 1 sun irradiation with a solar simulator (Newport 94043A) by putting the rGOHMs on a polystyrene foam inserted with water pumps in a container with a water volume height of 3 cm, or floating the rGOHMs on water surface in containers with water volumes of 3, 4, and 5 cm heights. The container was insulated by a polystyrene foam to prevent the heat conducted to the environment. Once the light was on, the mass change was immediately recorded by using an electronic balance with an accuracy of 0.1 mg connected to a computer for transferring the date of the real‐time mass change to calculate the evaporation rate and efficiency of solar steam generation. The surface temperatures of hydrogels were measured using an IR camera (Fluke), and the temperatures at different positions of water were measured using three thermocouples. During experiments, the ambient temperature and humidity were kept at ≈30 °C and ≈40%, respectively.

## Conflict of Interest

The authors declare no conflict of interest.

## Supporting information

Supporting Information

Supplemental Video 1

Supplemental Video 2

Supplemental Video 3
