# Supplementary material for: Stable Self‐Floating Reduced Graphene Oxide Hydrogel Membrane for High Rate of Solar Vapor Evaporation under 1 sun
Source: Glob Chall. 2020 Sep 28;5(1):2000053. doi: 10.1002/gch2.202000053 (PMC7788581; doi:10.1002/gch2.202000053)
Supplement: Supplementary file 1 — Supporting Information [file GCH2-5-2000053-s001.pdf]

© 2020 Wiley-VCH GmbH

Global Challenges
Open Access

Supporting Information

for *Global Challenges*, DOI: 10.1002/gch2.202000053

Stable Self-Floating Reduced Graphene Oxide Hydrogel Membrane for High Rate of Solar Vapor Evaporation under 1 sun

Pengyu Zhuang, Duo Li, Ning Xu, Xiaoqiang Yu, and Lin Zhou**

Supporting Information

Stable self-floating reduced graphene oxide hydrogel membrane for high rate of solar vapor evaporation under one sun

Pengyu Zhuang[1,2,†], Duo Li[1,†], Ning Xu[1],Xiaoqiang Yu[2,*], Lin Zhou[1,*]

[1]National Laboratory of Solid State Microstructures, College of Engineering and Applied Sciences, School of Physics, Key Laboratory of Intelligent Optical Sensing and Integration, Ministry of Education, Nanjing University, Nanjing 210093, P. R. China.

[2]School of Physics, Southeast University, Nanjing 211189, China

†These authors contributed equally to this work.

*Email: xqyu@seu.edu.cn, linzhou@nju.edu.cn

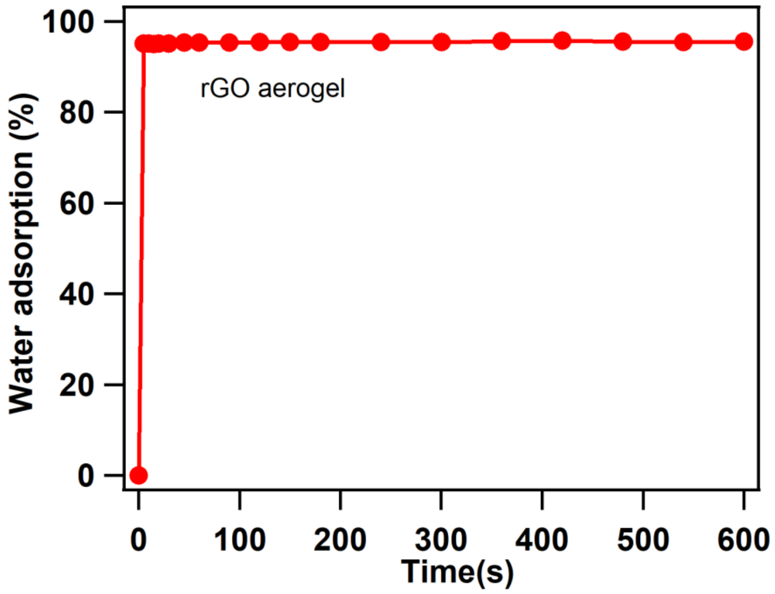

Fig. S1. Water adsorption of rGO aerogel as a function of time by floating it on water surface.

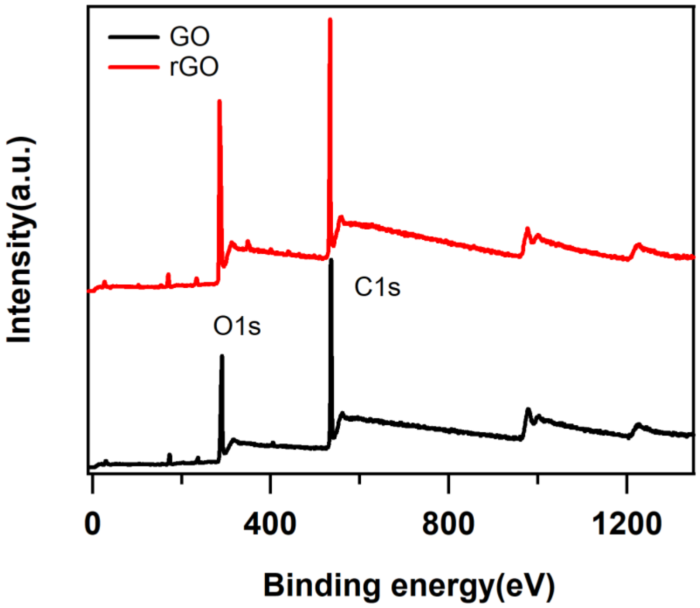

Figure S2. XPS general spectra of GO and rGO aerogels.

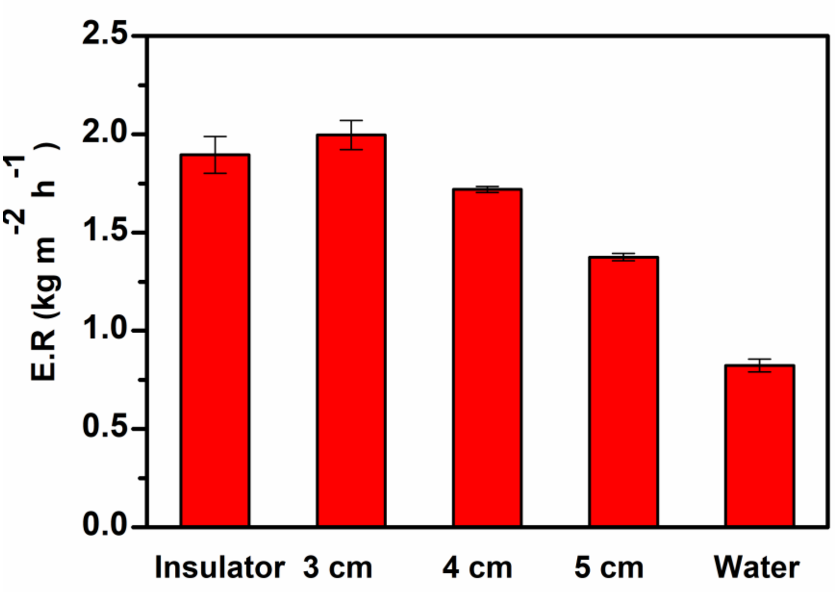

Fig. S3. The absolute averaged evaporation rates of solar steam devices under 1 sun.

Table S1. Summary of solar steam generation performance of graphene-based absorbers with both high evaporation rates and solar-to-heat conversion efficiencies under 1 sun.

Materials	Evaporation rates(kg m^{-2} h^{-1})	Absorption(%)	Efficiency(%)	Ref.

GO film	1.45	~ 94	80	[1]
rGO-SA-CNT aerogel	1.62	92	83	[2]
3D-CG-GN	1.25	97.2	85.6	[3]
h-G foam	1.7	~ 95	93.4	[4]
VA-GSM	1.62	~ 98	86.5	[5]
3D-graphene foam	1.3	~ 97	87	[6]
Graphene-based hive	2.13	~ 97.5	~ 94	[7]
rGO-SA-cellulose aerogel	2.25	~ 97	88.9	[8]
PAN/GO membrane	2.27	-	92.6	[9]
Biochar composite membrane	1.65	96	90.5	[10]
MoCC-chitosan hydrogel	~2.19	-	96.15	[11]
PVA-rGO hydrogel	~2.5	~ 98	~ 95	[12]
PVA hydrogel-AC	~2.6	~ 98	~ 91	[13]
$PP_{0.1}CS_{1.5}$ aeragel	1.78	96	-	[14]
CACW	2.22	93.3	93.2	[15]
rGOHM	2.33	~ 98	-	This work

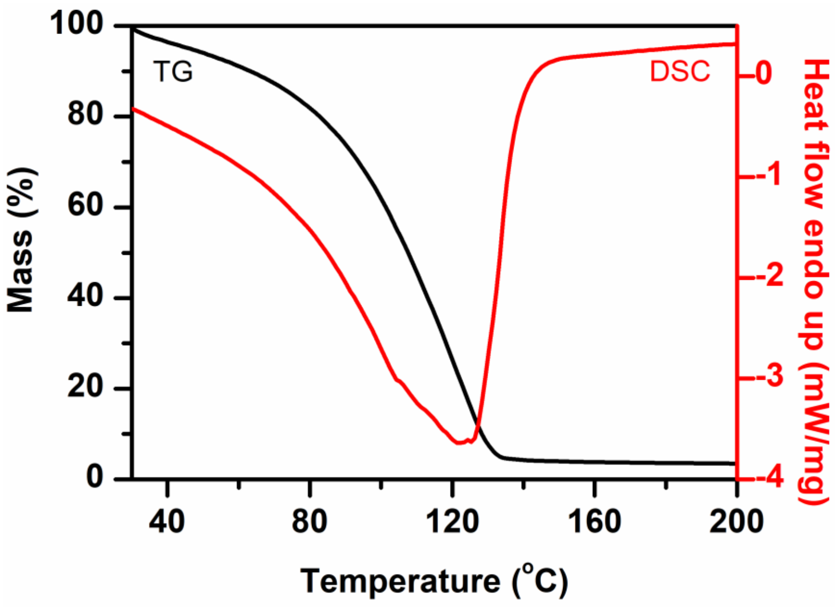

Fig. S4. TG-DSC thermograms for heating rGOHM.

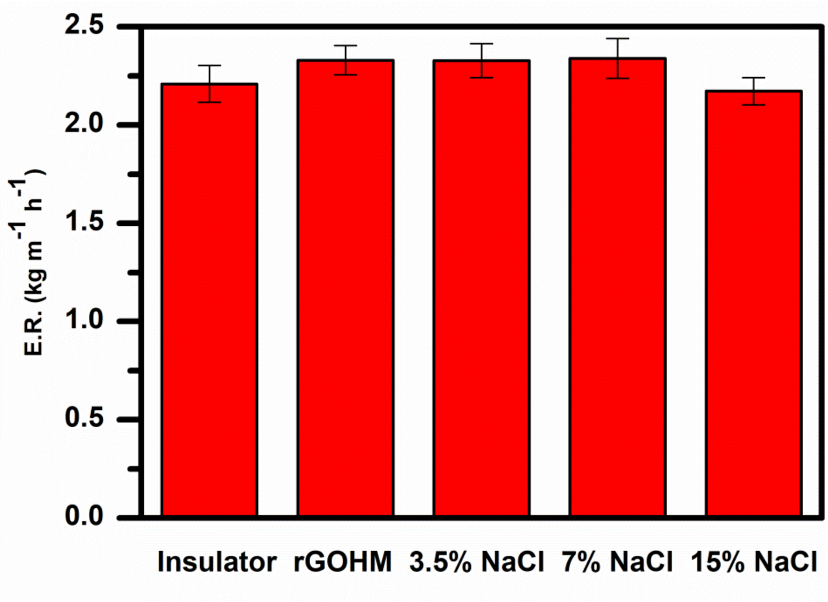

Fig. S5. The total evaporation rates of rGOHMs with/without insulators and NaCl solutions of different concentrations by considering the environment influences.

References

[1] X. Li, W. Xu, M. Tang, L. Zhou, B. Zhu, S. Zhu, J. Zhu, *Proc. Natl. Acad. Sci. U S A* 2016, **113**, 13953.

[2] X. Hu, W. Xu, L. Zhou, Y. Tan, Y. Wang, S. Zhu, J. Zhu, *Adv. Mater*. 2017, 29, 1604031.

[3] Y. Li, T. Gao, Z. Yang, C. Chen, W. Luo, J. Song, E. Hitz, C. Jia, Y. Zhou, B. Liu, B. Yang, L. Hu, *Adv. Mater*. 2017, **29**, 1700981.

[4] H. Ren, M. Tang, B. Guan, K. Wang, J. Yang, F. Wang, M. Wang, J. Shan, Z. Chen, D. Wei, H. Peng, Z. Liu, *Adv. Mater*. 2017, **29**, 1702590.

[5] P. Zhang, J. Li, L. Lv, Y. Zhao, L. Qu, *ACS nano* 2017, **11**, 5087.

[6] Y. Yang, R. Zhao, T. Zhang, K. Zhao, P. Xiao, Y. Ma, P.M. Ajayan, G. Shi, Y. Chen, *ACS nano* 2018, **12**, 829.

[7] X. Song, H. Song, S. Wang, J. Liu, L. Zhou, J. Xu, K. Chen, *J. Mater. Chem. A*

2019, **7**, 26496.

[8] D.P. Storer, J.L. Phelps, X. Wu, G. Owens, N.I. Khan, H. Xu, *ACS Appl. Mater. Interfaces* 2020, **12**, 15279.

[9] L. Wang, C. Liu, H. Wang, Y. Xu, S. Ma, Y. Zhuang, W. Xu, W. Cui, H. Yang, *ACS Appl. Mater. Interfaces* 2020, **12**, 24328.

[10] S. Zhang, L. Zang, T. Dou, J. Zou, Y. Zhang, L. Sun, *ACS omega* 2020, **5**, 2878.

[11] F. Yu, Z. Chen, Z. Guo, M.S. Irshad, L. Yu, J. Qian, T. Mei, X. Wang, *ACS Sustain. Chem. Eng*. 2020, **8**, 7139.

[12] X. Zhou, F. Zhao, Y. Guo, Y. Zhang, G. Yu, *Energ. Environ. Sci.* 2018, ***11***, 1985.

[13] Y. Guo, F. Zhao, X. Zhou, Z. Chen, G. Yu, *Nano Lett*. 2019, **19**, 2530.

[14] Y. Gu, X. Mu, P. Wang, X. Wang, J. Liu, J. Shi, A. Wei, Y. Tian, G. Zhu, H. Xu, J. Zhou, L. Miao, *Nano Energy*, 2020, **74**, 104857.

[15] X. Mu, Y. Gu, P. Wang, J. Shi, A. Wei, Y. Tian, J. Zhou, Y. Chen, J. Zhang, Z. Sun, J. Liu, B. Peng, L. Miao, *Solar RRL*, https://doi.org/10.1002/solr.202000341.
